# Impact of the built environment on stroke inpatient rehabilitation

**DOI:** 10.12968/bjnn.2023.19.Sup2.S19

**Published:** 2023-04-02

## Abstract

Guidance recommends that people with disability following stroke should receive rehabilitation in a dedicated stroke inpatient unit. Previous research has indicated that aspects of the built environment in inpatient settings can impact on patient wellbeing and experience. This article evaluates and discusses the findings of a recent systematic review that explores the effect of environmental and design factors on stroke rehabilitation.

## Introduction

Stroke is the second cause of death and a leading cause of disability worldwide and presents a major global burden (Katan and Luft, 2018). Over 100,000 people suffer strokes each year in the UK, many of whom are left with long-term effects and disabilities (Stroke UK, 2018). Many stroke patients require rehabilitation, a multidimensional programme of supervised activity which aims restore or improve physical and/or physiological function and quality of life (NICE, 2016). The National Institute for Health and Care Excellence guidance on rehabilitation for stroke patients recommends that people with disability after stroke should receive rehabilitation in a dedicated stroke inpatient unit (NICE, 2016). Previous research has indicated that aspects of the built environment in inpatient settings can impact on patient wellbeing and experience (Salonen et al., 2013; Dijkstra et al., 2006). For example, the presence of windows and a natural view have been shown to have a positive effect on delirium, sleep and length of stay in inpatients and better levels of sunlight have been shown to have positive effect on length of stay, mortality rate, and perceived stress and pain (Dijkstra et al., 2006). Since there is currently a move towards reconfiguring stroke care into specialist centralised centres in England (Iacobucci, 2019) it is important to consider how aspects of the built environment of inpatient settings can be used to enhance the rehabilitation outcomes, and the experience, of stroke patients. A recent systematic review by Lipson-Smith et al., 2021 aimed to identify existing design evidence and the impact of the built environment of inpatient stroke rehabilitation facilities on outcomes and patient, staff and family experience.

## Aim of commentary

This commentary aims to critically appraise the methods used within the review by Lipson-Smith et al., 2021 and to discuss the findings of the systematic review in the context of clinical practice.

## Methods

The authors carried out a search across the following databases from 2000 to November 2020: Ovid MEDLINE, Scopus, Web of Science and CINAHL. The search included key terms for stroke and the built environment. However, terms were only searched for in the title field meaning that the search was not highly sensitive, so it is possible that relevant papers could have been missed. The database searches were supplemented by checking reference lists of included articles, relevant systematic reviews and theses, key journals (Health Environments Research & Design), and the websites of relevant organisations (the Centre for Healthcare Design). The authors limited the search by date as they suggested that not many (or no) relevant papers would have been published before 2000. The search was limited to English language but no justification for this was provided. The inclusion criteria were outlined in a table. Quantitative, qualitative or mixed-methods studies involving adult stroke survivors, their families/carers and/or staff, and with detailed information about the built environment of inpatient settings, were included. A comprehensive screening process and assessment of quality (using the Mixed Methods Appraisal Tool (MMAT)) was undertaken by two reviewers independently. Data were extracted and then categorised by a single reviewer and the categories were reviewed by the wider review team. A narrative synthesis was conducted, structured around the categories identified.

## Results

18 studies were included in the review (24 articles). Of these, half were qualitative studies, and the rest were non-randomised quantitative studies or mixed methods studies and one randomised controlled trial. In the majority of studies (n=18), the outcomes and/or experiences of patients were explored as opposed to those of staff or family/carers.

Although the methodological quality was found to vary across the studies included, more than half the studies were judged to be of higher quality (MMAT score 5 or 4) with the qualitative studies having a higher median score (median MMAT score=5) than the non-randomised quantitative (median MMAT score = 4) or mixed methods studies (median MMAT score = 2). The most common criteria not achieved was the MMAT item related to study participants not being representative of the target population.

A narrative synthesis of the findings was conducted and structured around five outcome categories: patient clinical outcomes; patient activity; patient emotional wellbeing; patient and/or staff safety; and staff clinical practice and efficiency.

### Patient clinical outcomes

Six studies discussed patient clinical outcomes. One random controlled trial found that patients who had access to enriched inpatient environments reported improved self-care and mobility at discharge, and better health post-discharge compared with control. One nonrandomised controlled trial found fewer adverse events, such as worsening of symptoms, for patients experiencing enrichment compared to usual care. Two qualitative studies suggest that enhanced environments may facilitate improving patient outcomes. The remaining one quantitative and one qualitative papers were not discussed.

### Patient Activity

Thirteen studies reported on physical, cognitive and social activities. Two non-random controlled trials involving patients found that enriched environments and/or access to communal areas resulted in increased patient activity compared to usual care. Two mixed methods and one qualitative study indicated that attractiveness and ease of navigation of communal rooms is likely to be important in promoting the use and benefit of these spaces.

### Patient emotional wellbeing

Nine studies explored emotional wellbeing. One random controlled trial found that patients with access to communal areas had reduced levels of depression, stress and anxiety compared to patients without access. One non-random controlled trial found no difference in depression or anxiety between an old rehabilitation ward compared to an enhanced rehabilitation ward with improved lighting and more colour. Five qualitative studies identified that access to communal areas was perceived to reduce boredom and loneliness and can be perceived to promote patient empowerment.

In four qualitative studies, patients and staff identified that flexibility of spaces, connection to nature/the outside world, privacy and control over the space, aesthetics (such as lighting, noise levels, etc.,) and ease of navigation and access were possible environmental factors contributing to emotional wellbeing of patients.

### Patient and/or staff safety

Three of the studies involving staff and patients addressed safety. Two qualitative studies identified that environmental aspects such as accessibility, minimising manual handling, sightlines between staff and patients, lighting and noise, and consideration of potential barriers such as uneven flooring and obstacles, were perceived to improve safety of patients, staff and visitors/family.

### Staff clinical practice and efficiency

Clinical practice and/or efficiency was mentioned in ten of the studies. One nonrandomised controlled study found that there was no evidence that staff workload increases after activity areas were introduced. Two qualitative studies identified that staff opinions varied as to whether the provision of patient communal areas increases or decreases clinical workload. One mixed method study found the design of the stroke units observed did not appear to foster multi-professional staff teamwork. Another qualitative study concluded that suitability of treatment spaces could impact on treatment decision-making by staff (because therapists adapted to the space they had available).

## Commentary

Using an adapted version of the Joanna Briggs Institute Critical Appraisal Tool for systematic reviews (Joanna Briggs Institute, 2020), 8 out of 9 criteria were judged to be satisfactory for this review. The criteria which weren’t achieved were regarding the methods of synthesis and the inclusion criteria. Where mixed method studies were used it was unclear which part of the mixed method design supported each statement which makes it difficult to identify the quality of evidence underpinning the statement. Where nonrandomised trials were used it was unclear if the control comparison was the difference between intervention and control at the end of study or the difference between before and after. In the former it is important to indicate the baseline comparison as due to the non-randomisation there is a chance of unequal groups at baseline. Furthermore, where qualitative studies were combined the methods of synthesis were not transparent. Therefore, the findings from this review should be viewed with caution.

Additionally, as highlighted in the Methods section, the data were extracted and categorised by a single reviewer which could be viewed as a further methodological limitation of the review since it has been shown that single data extraction results in more errors than double data extraction (Buscemi et al., 2006). The quality assessment done by the reviewers also found that many of the included studies in the review had a low MMAT score for study participants being representative of the target population. These limitations should be taken into consideration, and the population investigated should be taken into account, when interpreting the findings of the review.

Regarding practice the findings from this review suggest that it is important to consider the environment and its possible impact on patient outcomes. This can be facilitated by taking the following factors into consideration. When developing a rehabilitation environment, it is beneficial to make the environment engaging for patients providing them with books, games, and activities of their choice. It is important that patients have a clear choice of activities which would be engaging for them (Royal College of Physicians, 2016; NICS, 2012). However, as highlighted in this review, and a recent Cochrane review (Qin et al., 2021), there is insufficient evidence to suggest a definitive set of activities to enhance rehabilitation in these environments. Therefore, activities should be things which patients want to engage in (Janssen et al., 2022) for example, electronic games (Ortiz-Huerta et al., 2018; Saeedi et al., 2021), virtual reality (Charles et al., 2020; Choi et al., 2018), card/puzzle games (Hung et al., 2016) and board games (Caballero-Coulon et al., 2007).

Nearly a third of patients experience a mood disorder (such as depression or anxiety) following a stroke (Mitchell et al., 2017). Weak evidence from this review suggests that access to communal areas can help reduce levels of depression, stress, and anxiety in inpatients. Additional limited evidence suggests that environments should be nature-based and flexible in design providing dynamic lighting and privacy when required, to enhance patient wellbeing. There was also low-level evidence from the review to suggest that ease of access may be an important moderating factor in the use of communal spaces. Many stroke patients have impaired mobility with around half of patients unable to walk independently in the first 3-5 days following a stroke (Louie et al., 2022). If possible, facilities should have compact layouts since longer distances between different hospital areas have been shown to hinder patient mobility (Kevdzija and Marquardt, 2022). Consideration should be given to the complexity and design of the building layout as stroke patients often have impaired navigation skills (Claessen et al., 2017; Kevdzija, 2022). Furthermore, these environments should be assessed for safety to ensure that uneven flooring and obstacles are minimised. Flooring such as rugs and thick carpets should be avoided (Rosen et al., 2013). In regard to staffing requirements there was no evidence found by the review to suggest that additional staffing would be required if communal/activity areas were provided.

Further research into all aspects of the built environment for stroke patients is needed to strengthen the level of evidence in this area. Investigation into how the built environment impacts on the experiences of family and visitors also needs to be explored as there is currently little evidence for this group. Research into targeted rehabilitation activity assessing patient clinical outcomes, in addition to exploratory environmental considerations, could be undertaken. To effectively grow the research field studies conducted in other healthcare systems and cultural settings would add to our knowledge of the impacts of the built environment.

## CPD reflective questions

What is the strength of the evidence presented in this review?

Do you think there is anything practitioners can do in practice to influence the physical space of clinical environments?

What further research is required on this topic?

## Key Points

1. Evidence from the review indicates that aspects of the built environment in stroke inpatient settings, such as enrichment, access to communal areas, attractiveness and flexibility of spaces and ease of navigation, may impact on patient outcomes and experience.
2. There is low level evidence that access to communal areas increases patient activity and can help reduce depression, anxiety and stress in patients following stroke.
3. Further research is needed on all aspects of the built environment to strengthen the evidence in this area.
